# Lymphadenectomy promotes tumor growth and cancer cell dissemination in the spontaneous RET mouse model of human uveal melanoma

**DOI:** 10.18632/oncotarget.6326

**Published:** 2015-11-02

**Authors:** Yeo Kim Pin, Karen Khoo, Muly Tham, Tan Karwai, Thiam Chung Hwee, Anne-Laure Puaux, Meow Ling Cindy Phua, Masashi Kato, Veronique Angeli, Jean-Pierre Abastado

**Affiliations:** ^1^ Department of Microbiology, Immunology Programme, Life Science Institute, Yoon Loo Lin School of Medicine, National University of Singapore, Singapore; ^2^ Singapore Immunology Network, BMSI, A-STAR, Singapore; ^3^ Department of Clinical Research, Singapore General Hospital, Singapore; ^4^ Department of Occupational and Environmental Health, Nagoya University Graduate School of Medicine, Nagoya, Japan; ^5^ Present address: Institut de Recherches Internationales Servier, Suresnes Cedex, France

**Keywords:** lymphadenectomy, uveal melanoma, inflammation

## Abstract

Resection of infiltrated tumor-draining lymph nodes (TDLNs) is a standard practice for the treatment of several cancers including breast cancer and melanoma. However, many randomized prospective trials have failed to show convincing clinical benefits associated with LN removal and the role of TDLNs in cancer dissemination is poorly understood. Here, we found in a well-characterized spontaneous mouse model of uveal melanoma that the growth of the primary tumor was accompanied by increased lymphangiogenesis and cancer cell colonization in the LNs draining the eyes. But, unexpectedly, early resection of the TDLNs increased the growth of the primary tumor and associated blood vessels as well as promoted cancer cell survival and dissemination. These effects were accompanied by increased tumor cell proliferation and expression of phosphorylated AKT. Topical application of a broad anti-inflammatory agent, Tobradex, or an oral treatment with cyclooxygenase-2 specific inhibitor, Celecoxib, reversed tumor progression observed after complete lymphadenectomy. Our study confirms the importance of tumor homeostasis in cancer progression by showing the enhancing effects of TDLN removal on tumor growth and cancer cell dissemination, and suggests that TDLN resection may only be beneficial if used in combination with anti-inflammatory drugs such as Tobradex and Celecoxib.

## INTRODUCTION

Lymph nodes (LNs) are strategically positioned and highly organized structures that provide optimal interactions of components of the immune system to induce immune response against invading pathogens and tumors. The relationship between tumors and the immune system is complex and it is now appreciated that the immune system has a dual role in cancer. Indeed, it does not only suppress tumor growth by killing cancer cells or impeding their growth but also support tumor progression by shaping tumor immunogenicity or promoting immunosuppressive conditions within the tumor environment [1]. The concept of “cancer immunoediting” is an attempt to integrate this dual host-protective and tumor-promoting actions of immunity on the development and progression of cancer [1].

Integrity of lymphatic vessels (LVs) and LNs is not only critical for proper immunologic functions but also for maintaining fluid balance by draining interstitial fluid, lipids, cytokines, chemokines and growth factors from tissues back to the blood circulation [2, 3]. Notably, each LN drains a define territory and all tissues, with a few exceptions, are drained by LNs. Consequently, each tumor has its own tumor-draining lymph node(s) (TDLNs) [4, 5]. Lymphatic fluid drained away from the primary tumor may contain tumor cells or tumor-derived factors. The tumor cells may then form secondary tumors within the “sentinel” LN which may subsequently colonize the next draining LN. Tumor cells transported by lymphatics can also reach the blood circulation and then disseminate to other distant organs to form metastasis [6]. Analysis of the TDLN for the presence of metastasis has been recognized as a critical clinical staging system in the metastatic process and surgical removal of TDLN is now widely used in clinic as a means of determining future treatment [7]. Although the involvement of the sentinel LN is an important prognostic factor, complete lymphadenectomy (CLND) does not seem to promote survival. Many clinical trials reported to date showed little if any survival benefit of CLND, even after several decades of follow up [8–13]. The role of TDLNs in tumor growth and dissemination is poorly understood, and therefore the consequences of CLND need further investigations.

MT/ret mice (refer to as RET mice from now) are immune-competent mice transgenic for the human *RET* oncogene which is specifically expressed by melanocytes [14, 15]. In this mouse model of human melanoma, tumor develops in the uvea (choroid, ciliary body or iris), a tissue rich in melanocytes and relatively protected from the immune system. Unlike transplanted tumor models, RET mice spontaneously develop clinically detectable uveal melanomas at three to eight weeks of age, followed by a rapid and progressive metastatic process [16]. Our previous work showed that cancer cells disseminate as early as three weeks after birth [16]. The disseminated cancer cells remain dormant for months before developing into cutaneous or visceral metastases. We also showed that in a given mouse, metastatic tumors share a common clonal origin. The stepwise evolution of melanoma in RET mice recapitulates the natural history of disease progression in cancer patients, underlining the significance and suitability of this melanoma model to study the effect of CLND on tumor growth and dissemination.

In this study, we first identified LNs that drain uveal tumors in the RET mouse model in order to perform CLND. Unexpectedly, we found that CLND promoted the growth of primary uveal tumor nodule, cancer cell dissemination and metastasis. These effects were associated with increased proliferation and survival of tumor cells and phosphorylation of AKT which were reversed by treatments with anti-inflammatory drugs.

## RESULTS

### Cervical lymph nodes drain uveal tumors

**Figure 1:**
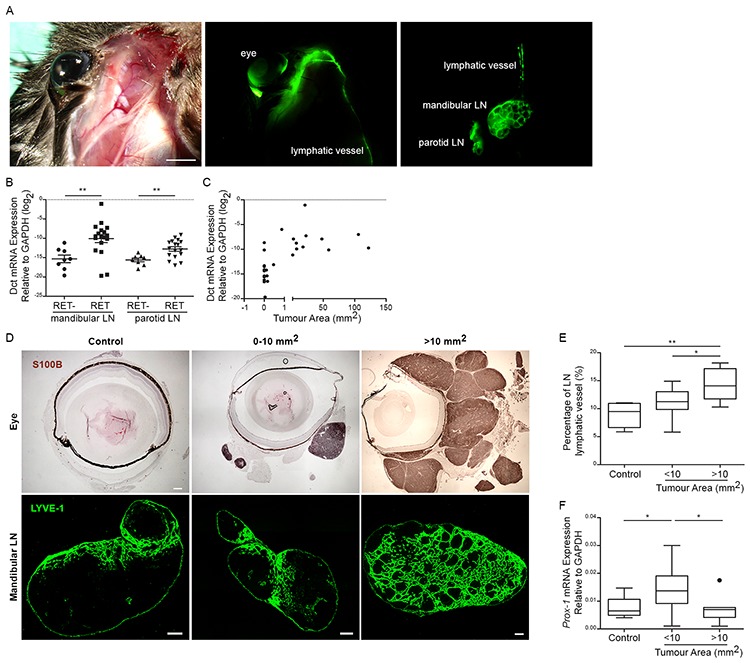
Cervical lymph nodes drain uveal tumors **A.** FITC-conjugated dextran was injected intra- or peri-ocularly into RET mice. LNs and LVs connecting the eyes to mandibular and parotid LNs were recognized by positive FITC signal (Green). Scale bar = 2 mm. **B.** Dct expression was measured by qRT-PCR in mandibular and parotid LNs of RET mice and non-transgenic control littermates (RET-). Each point represents one LN. two-tailed Mann-Whitney test; ** *p* value < 0.01 (*n* = 6–9 mice). **C.** Correlation of Dct expression in mandibular LN was plotted as a function of primary tumor area (mm^2^). Tumor area was measured by counting the sum of S100B^+^ tumor cells from 5 representative primary tumor cross-sections. Spearman's correlation r = 0.6529; ***p value < 0.001 (*n* = 19 mice). **D.** Top image panels: Eye tumors stained with S100B antibody (brown) and size of tumor areas are indicated as mm^2^. Scale bar = 300 um. Bottom image panels: LVs in the mandibular LNs stained with Lyve-1 antibody (green). Scale bar = 200 um. **E.** LV area was measured as mean fraction of Lyve-1^+^ staining from LN. Total tumor area was measured by counting the sum of S100B^+^ tumor cells from 5 representative primary tumor cross-sections. 1-way Anova; * *p* value < 0.05 (*n* = 5–6 mice). **F.** qRT-PCR analysis of *Prox-1* transcripts was measured in mandibular LNs of RET mice and control littermates. 1-way Anova; *p value < 0.05 and **p value < 0.01 (*n* = 6–12 mice).

Although uveal melanomas metastasize predominantly by hematogenous spread, they can occasionally metastasize to the draining mandibular or parotid LNs and intraocular injection of tumor cells can result in cell dissemination to TDLNs [17–20]. To verify that these LNs drain the primary tumor in RET mice, FITC-conjugated dextran was injected peri- or intra-ocularly and cervical region was imaged 20 mins later. Fluorescent signal was detected in both ipsilateral mandibular and parotid LNs as well as the corresponding efferent LV (Figure 1A). Immunofluorescent staining of tumor-bearing eyes from RET mice also indicated the presence of peri-tumoral LVs while intra-tumoral LVs were rare (Supplementary Figure S1). Next, we evaluated the presence of tumor antigens in these TDLNs from RET mice and non-transgenic littermates. Ectopic expression of the melanocytic gene, daupachrome tautomerase (Dct, an enzyme involved in melanin synthesis), is a sensitive and reliable marker for cancer cell dissemination in RET mice [16]. *Dct* expression was significantly higher in the mandibular and parotid LNs of tumor-bearing mice as compared to non-transgenic littermates (Figure 1B) and correlated with primary tumor size (Spearman's correlation *r* = 0.65; *p* < 0.0001) (Figure 1C). Staining for LV endothelial hyaluronan receptor-1 (Lyve-1), a specific marker of LVs revealed extensive lymphangiogenesis in TDLNs from mice with large uveal tumor (>10 mm^2^) (Figure 1D–1E). Expression of *Prox-1*, a transcription factor expressed by lymphatic endothelial cells, was increased in the mandibular LNs draining small tumor (<10 mm^2^) but returned to baseline level in larger tumors (>10 mm^2^) (Figure 1F).

### CLND stimulates the growth of blood vessels, primary tumors and metastatic tumors

**Figure 2:**
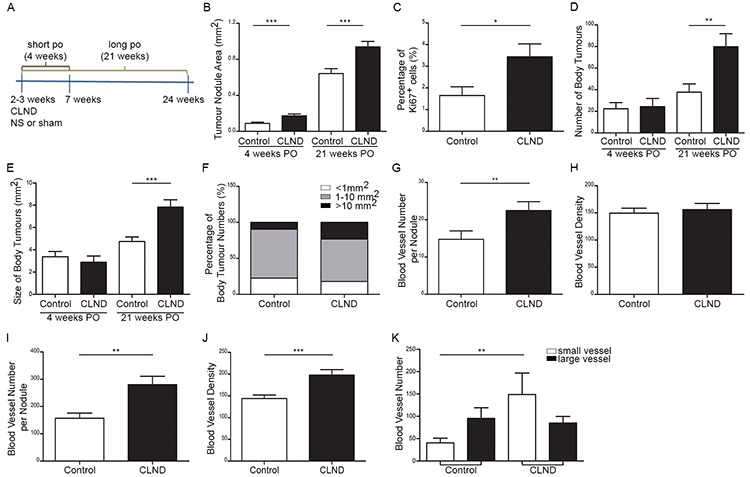
CLND stimulates growth of the blood vessels, primary tumors and metastatic tumors **A.** Schematic diagram depicting CLND experimental plan. 2–3 week-old RET mice was subjected to bilateral complete LN resection. Sham control was performed under similar surgical procedure without removing the LNs. Necropsy was performed 4 and 21 weeks-post operation. **B.** Quantification of the area of tumor nodules within the orbit of the eye 4 and 21 weeks after CLND. two-tailed Mann-Whitney test, *** *p* value < 0.0001 (*n* = 5–20 mice). **C.** The percentage of Ki67^+^ cells in the tumor nodules 4 weeks after CLND; two-tailed Mann-Whitney test; * *p* value < 0.05 (*n* = 5–6 mice). **D–E.** Number and area of metastases present in mice 21 weeks after CLND; two-tailed Mann-Whitney test, ** *p* value < 0.01 and *** *p* value < 0.001 (*n* = 10–11 mice). **F.** Body metastases were segregated into three different sizes (<1 mm^2^, 1–10 mm^2^ and > 10 mm^2^). **G–J.** BVs in primary tumor cross-sections were identified by CD31 immunostaining 4 and 21 weeks after CLND. Number of BV per tumor nodule was measured by counting all intra-tumoral BVs in each tumor nodule while BV density was the value of all intra-tumoral BVs normalized by corresponding tumor nodule area; two-tailed Mann-Whitney test; ** *p* value < 0.01, *** *p* value < 0.001. **(K)** BVs of long post survival group were categorized into two groups based on small (<2000 pixel) or large BV (>2000 pixel); 1-way anova; ** *p* value < 0.01.

Having established that melanoma cells can disseminate to the cervical LNs, we next assessed the effects of CLND on primary tumor growth. Two- to three-week old mice were subjected to complete bilateral resection of mandibular and parotid LNs and analysed 4 and 21 weeks post-operation (Figure 2A). Non-surgery (NS) control and sham control (similar surgical procedure without removing the TDLNs) were pooled together and defined as control group in subsequent experiments since there was no significant difference in the parameters assessed herein (Supplementary Figure S2A–S2C). The primary uveal tumors are often multi-nodular and the individual tumor nodules isolated 4 or 21 weeks post-operation were significantly larger in CLND-resected animals than the controls at both time-points (Figure 2B). Significant increase in the percentage of Ki67-positive proliferating cells was also observed 4 weeks after surgery (Figure 2C). Furthermore, while there was no difference in both number and size of body metastases 4 week after surgery, a 53% increase in the number of body metastases was observed in the 21-week post-operated CLND group when compare to controls (Figure 2D). The size of body metastases was also significantly larger in the CLND-resected group than the controls (Figure 2E). Twenty one weeks after CLND, the number of large tumors (>10 mm^2^) increased by 2.5 fold compared to control group (Figure 2F). The role of blood vessels (BVs) in tumor growth and hematogenous dissemination [21] prompted us to assess whether enhanced angiogenesis may account for the increase in primary tumor growth and cancer cell dissemination following CLND. The number of BV per tumor nodule in the primary tumor 4 weeks after CLND increased significantly while BV density did not differ between the two groups (Figure 2G–2H). By 21 weeks post-surgery, both the BV number and density in primary tumors after CLND were augmented significantly (Figure 2I–2J). Notably, the increase in smaller BVs mostly accounted for the overall increase in BVs in the long post-operative group (Figure 2K). These data shows that CLND during early tumorigenesis not only can stimulate and enhance growth of the primary tumor resulting in overall larger tumor mass in the long term, but also can exacerbate cancer cell dissemination. Furthermore, CLND-induced tumor growth is associated with increased angiogenesis.

### Topical tobradex and oral celecoxib impede CLND-induced angiogenesis, tumor growth and metastasis

**Figure 3:**
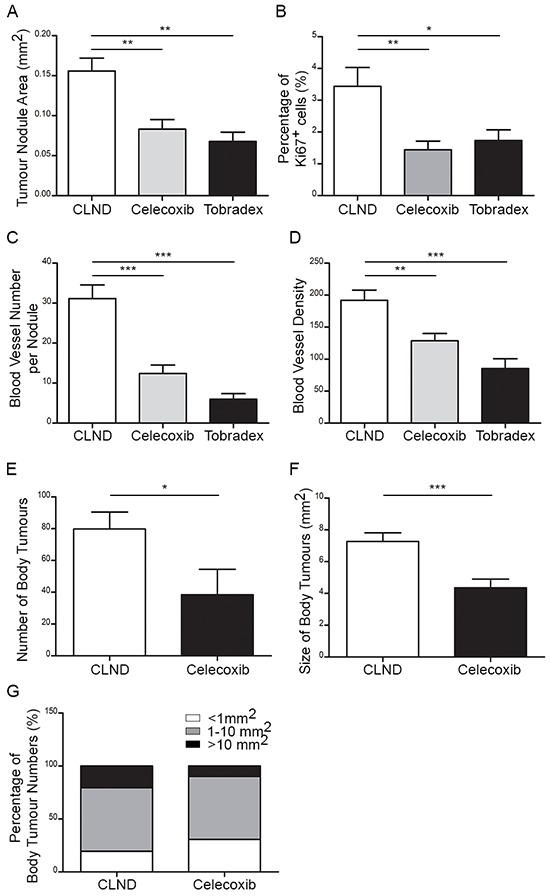
Topical Tobradex and oral Celecoxib attenuate CLND-induced blood vessel growth, tumor growth and metastasis. 2–3 week-old RET mice that underwent CLND were treated or not with daily topical application of Tobradex or fed a diet containing Celecoxib for 4 or 21 weeks **A.** After 4 weeks of treatment, primary tumor nodule areas were measured. 1-way anova; ** *p* value < 0.01 (*n* = 7–14 mice). **B.** The percentage of Ki67^+^ cells in the tumor nodules 4 weeks after CLND with or without treatments; two-tailed Mann-Whitney test; * *p* value < 0.05 (*n* = 5–8 mice). **C–D.** Number of BVs per tumor nodule and density were measured. 1-way anova; ** *p* value < 0.01, *** *p* value < 0.001 (*n* = 6–12 mice). **E–F.** Number and area of body metastases were quantified. two-tailed Mann-Whitney; * *p* value < 0.05 and *** – value < 0.0001. **G.** Each body metastases were segregated into three different sizes (<1 mm^2^, 1–10 mm^2^ and > 10 mm^2^).

The microenvironment of many human and murine cancers is rich in pro-inflammatory cytokines and growth factors. This inflammatory environment is known to contribute to cancer progression [22]. Furthermore, LN removal is expected to promote and/or sustain inflammation at the upstream peripheral site by disrupting lymphatic drainage (Supplementary Figure S3) [23, 24]. Therefore, we next investigated whether anti-inflammatory drugs may reverse the effect of CLND. One group of RET mice received topical application of the potent anti-inflammatory drug, Tobradex, on the eyes for 4 weeks following surgery. Tobradex is an ophthalmic ointment containing tobramycin (antibiotic) and dexamethasone (anti-inflammatory) [25]. In another experimental group, RET mice were fed on a normal or Celecoxib-supplemented diet for 4 weeks following CLND. Celecoxib is a non-steroidal anti-inflammatory drug (NSAID) that selectively targets cyclooxygenase-2 (cox-2) and has been shown to be chemopreventive and chemoprotective [26]. In both Tobradex and Celecoxib-treated groups, primary tumor nodule growth was hindered 4 weeks after surgery compared to the non-treated CLND group (Figure 3A). Celecoxib and Tobradex treatments reduced the primary tumor area by 1.9- and 2.4-fold, respectively. This was further supported by a marked reduction in the percentage of proliferating uveal tumor cells in the treated groups (Figure 3B). In addition, BV number and density also decreased significantly in treated groups (Figure 3C–3D). For long term experiments, only Celecoxib-incorporated diet was given to RET mice after CLND because daily application of Tobradex for 21 weeks did not seem clinically relevant. Notably, Celecoxib supplementation for 21 weeks significantly reduced the number and size of metastatic tumors compare to untreated mice (Figure 3E–3F). Number of large tumors (>10 mm^2^) was also reduced by 2.2-fold after Celecoxib administration (Figure 3G). Together, these results show that short term treatments with Tobradex or Celecoxib following CLND potently control primary tumor and BV growth. Long term Celecoxib treatment strongly impedes metastatic tumor number and size, markedly hampering early tumor dissemination and metastasis induced by CLND.

### Uveal tumor cells express VEGF-A and VEGFR-1

**Figure 4:**
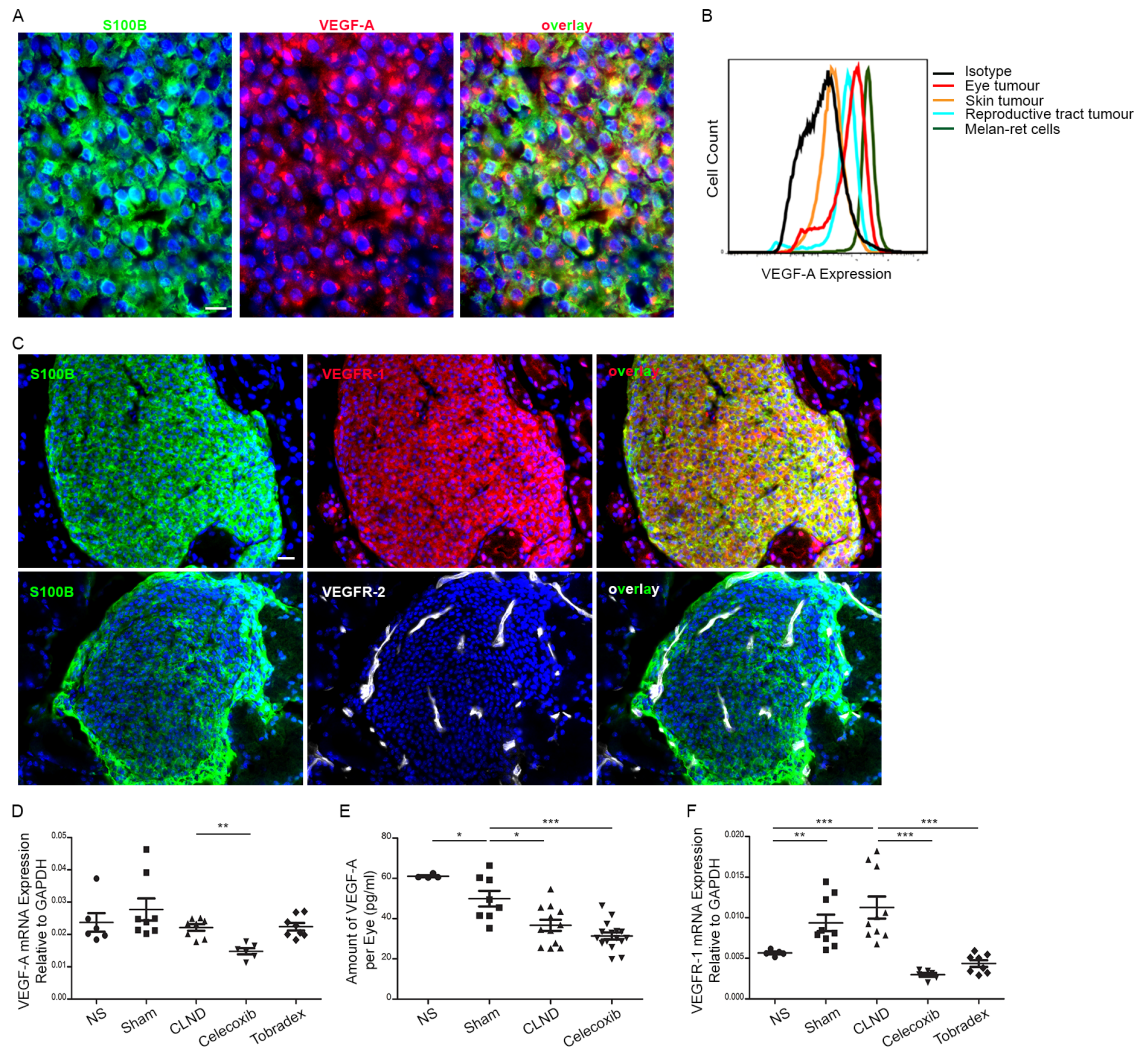
Uveal tumor cells express VEGF-A and VEGFR-1 **A.** Eye tumors cross-sections were immunostained with anti-S100B (green) and anti-VEGF-A (red) antibodies. Scale bar = 10 um. **B.** Intracellular expression of VEGF-A in tumor cells from uveal (red), skin (orange) or reproductive tract tumors (blue), and Melan-ret cell line (green). Cells immunostained for CD45^lo^CD31^lo^PDGFRα^lo^VEGF-A^+^ were evaluated by flow cytometry. **C.** Immunofluorescent staining for VEGFR-1 (top) or -R2 (bottom) in primary uveal tumor cross-sections. Scale bar = 20 um. **D.** Graph comparing the gene expression level of VEGF-A relative to GAPDH in primary uveal tumor from NS, sham, CLND, Celecoxib- and Tobradex-treated mice. Each point represents a tumor bearing eye (*n* = 3–8 mice). two-tailed Mann-Whitney; ** *p* value < 0.01. **E.** VEGF-A protein content in homogenates of NS, sham, CLND and Celecoxib-treated uveal tumors was examined by ELISA. Each point represents a tumor bearing eye (*n* = 5–16 mice). two-tailed Mann-Whitney; * *p* value < 0.05 and *** *p* value < 0.001. **F.** Quantitative RT-PCR analysis of VEGFR-1 in uveal tumors of NS, sham, CLND, Celecoxib- and Tobradex-treated mice. Each point represents a tumor-bearing eye (*n* = 3–8 mice). two-tailed Mann-Whitney; ** *p* value < 0.01 and *** *p* value < 0.001.

Since VEGF-A is one major factor for angiogenesis and tumor growth, we investigated whether VEGF-A could mechanistically explain the increased blood and tumor growth observed after CLND in RET mice. First, we confirmed that tumor cells in tumor-bearing eyes expressed VEGF-A by immunofluorescence (Figure 4A) and flow cytometry (Figure 4B). Notably, among the tumors derived from RET mice the eye tumors exhibited the highest levels of VEGF-A expression. ELISA measurement of supernatant from Melan-ret cells, a cell line derived from a RET tumor [27], further supported that these tumor cells secrete VEGF-A (data not shown). We next verified the expression of VEGF-A receptors, VEGFR-1 and -2 in tumor cells and tumor-associated BVs. Uveal tumor cells expressed VEGFR-1 while BV expressed VEGFR-2 (Figure 4C), suggesting that tumor cells and BVs can utilize VEGF-A for growth and/or survival. The expression of VEGFR-1 on tumor cells was further confirmed using Melan-ret cells (Supplementary Figure S4). Owing to the sensitivity of qPCR and ELISA techniques, NS and Sham data were not combined for these analyses. VEGF-A mRNA expression remain unchanged in non-treated groups, suggesting that CLND did not increase VEGF-A synthesis *de novo* (Figure 4D). Celecoxib, but not Tobradex, treatment reduced VEGF-A mRNA expression. In contrast, the amount of VEGF-A protein slightly decreased in sham control group and was further reduced in the CLND group (Figure 4E). Reduction in VEGF-A protein accompanied by unchanged VEGF-A synthesis after CLND led us to postulate that tumor cells and BVs may consume this factor for their growth. VEGFR-1 mRNA expression was significantly upregulated in sham control group and further increased after CLND whereas VEGFR-2 gene expression increased after CLND (Figure 4F and data not shown). Finally, both Celecoxib and Tobradex drastically reduced the synthesis of VEGFR-1 (Figure 4F).

### CLND-associated upregulation of activated AKT protein in uveal tumor cells is reversed by celecoxib and tobradex

**Figure 5:**
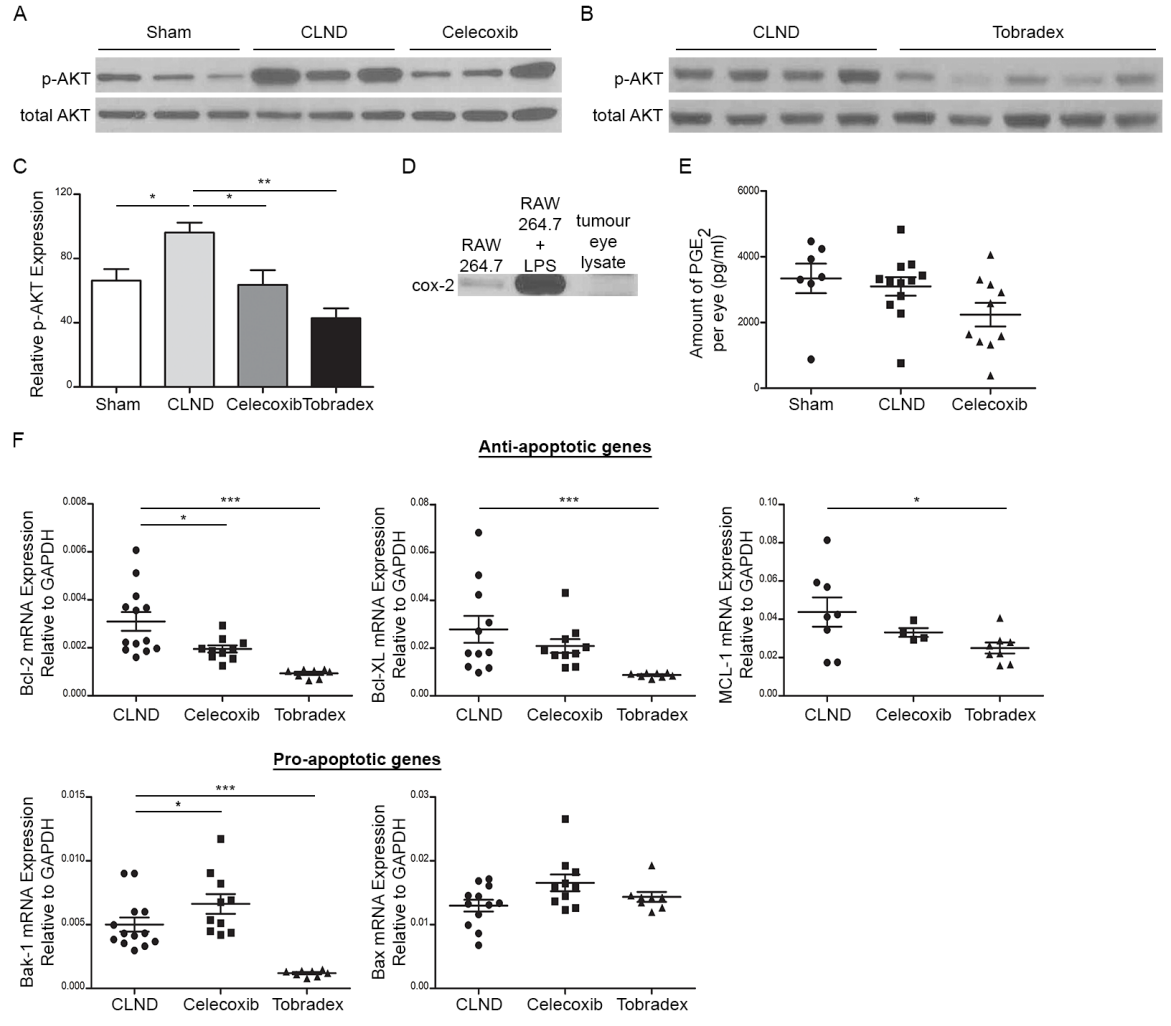
Celecoxib and Tobradex reduce CLND-induced upregulation of activated AKT proteins in uveal tumor Western blot analysis of phosphorylated and total AKT uveal tumor lysates in sham, CLND, Celecoxib **A.** and Tobradex **B.**. **C.** Relative protein expression was measured by densitometry analysis using Image J software and calculated as the percentage of phosphorylated AKT over total AKT protein expression. **D.** Western blot analysis of cox-2 protein in tumor-bearing eyes. RAW264.7 cells treated with or without LPS served as positive and negative control, respectively. **E.** PGE_2_ concentration from sham, CLND and Celecoxib-treated uveal tumor lysates was quantified by ELISA. Each point represents a tumor-bearing eye (*n* = 7–12 mice). two-tailed Mann-Whitney **F.** Quantitative RT-PCR analysis of anti-apoptotic genes (Bcl-2, Bcl-XL and MCL-1) and pro-apoptotic genes (Bak-1 and Bax) in tumor-bearing eyes of CLND, Celecoxib- and Tobradex-treated mice. Each point represents one tumor-bearing eye (*n* = 7–10 mice). two-tailed Mann-Whitney test; * *p* value < 0.05, *** *p* value < 0.001.

As we observed increased tumor cell proliferation and tumor growth in response to CLND, we decided to evaluate the downstream VEGF-A signalling pathways leading to phosphatidylinositol 3′-kinase (PI3K)/AKT and the Mitogen Activated Protein Kinase (MAPK) activation that are relevant to melanoma growth and survival [28–30]. Immunoblotting using phospho-specific antibodies revealed a significantly higher expression of pAKT while pERK1/2 expression remained unchanged in the tumor-bearing eyes of the CLND group as compared to control group (Figure 5A–5B and data not shown). Assessment of the effect of Celecoxib and Tobradex on pAKT revealed that both treatments significantly decreased pAKT in uveal tumor lysates (Figure 5A–5C). Cox-2 is constitutively overexpressed in many human premalignant, malignant and metastatic tumors and its upregulation promotes tumor cell proliferation, angiogenesis, invasion and metastasis [31–34]. We therefore evaluated if cox-2 and its metabolite, prostaglandin-E2 (PGE_2_), were expressed by uveal tumor cells from RET mice by Western blotting and ELISA, respectively. The macrophage cell line, RAW264.7, treated with or without LPS served as our positive and negative controls, respectively. Cox-2 protein was not detected in lysate from uveal tumors and PGE_2_ concentration did not differ significantly among sham, CLND and Celecoxib-treated groups (Figure 5D–5E) suggesting that Celecoxib's ability to reduce CLND-induced tumor growth is likely cox-2 independent. We, therefore, evaluated if there were any changes in anti- or pro-apoptotic gene expression in uveal tumors from treated- and non-treated CLND groups since mitochondrial apoptosis pathway can be induced by cox-2 inhibitors [35]. Tobradex potently inhibited the expression of anti-apoptotic genes including BCL-2, BCL-XL and MCL-1 while the expression of pro-apoptotic Bax and Bak-1 genes remained unchanged or decreased compared to the CLND group (Figure 5F). Celecoxib reduced anti-apoptotic BCL-2 expression while significantly increasing pro-apoptotic Bak-1 expression (Figure 5F). A decreased trend in BCL-XL and MCL-1 mRNA expression and increased trend in Bax expression level were also observed in Celecoxib-treated group. These data suggest that Tobradex and Celecoxib may interfere with the activation state of the kinase AKT and the balance between anti-apoptotic and pro-apoptotic genes.

## DISCUSSION

For most cancer types, metastasis remains responsible for more than 90% of cancer-related death. This emphasizes the need for a better understanding of underlying mechanisms of this process in order to control metastatic disease. Analysis of the metastatic status of TDLNs is of central prognostic significance and the removal of TDLNs is now commonly used as an important guide for subsequent post-operative therapy. However, our current data show in RET mice that CLND significantly promotes primary tumor growth and metastasis. This suggests that the LN metastases may not contribute significantly to the formation of metastases in distant organs and challenges the view of the involvement of LN metastasis in the metastatic cascade. Indeed, if established LN metastases serve as a cancer cell reservoir for metastasis in our model, the RET mice should have developed less clinically overt metastatic tumors after CLND. But instead, we found that CLND significantly increased metastatic outgrowth. Our results are in line with the idea that LN metastasis is more likely an indicator of metastatic progression [36] rather than a governor of metastatic development. It is also consistent with the lack of improved survival observed after lymphadenectomy in melanoma patients [8–13].

How might resection of LN contribute to tumor growth and cancer cell dissemination in our mouse model of melanoma? It is well known that cancer development and formation of metastasis are not entirely regulated by cancer-cell autonomous changes, but may be influenced, and possibly driven, by the cross-talk between tumor cells and their microenvironment which may include immune cells, stromal cells and extracellular matrix [37, 38]. Therefore, we propose that the removal of LN may promote the growth of primary tumor and cancer cell dissemination by stimulating a pro-tumoral environment. It is known that the microenvironment of many cancers in humans and experimental models is rich in pro-inflammatory cytokines and growth factors that contribute to cancer progression [22]. Impaired lymphatic drainage as a result of, for example, LN resection has been shown to compromise the transport of inflammatory cells and soluble mediators [3, 39, 40] and the resolution of certain inflammatory diseases [40–42]. Notably, secondary lymphedema, local inflammation and delayed wound healing have been reported in melanoma, breast and cervical cancer patients after lymphadenectomy [43–45]. Thus, the early removal of TDLNs in our cancer model may be sufficient to create local inflammation that stimulates the proliferation and survival of tumor cells, as well as angiogenesis which may also contribute to tumor growth. In support of this, we show that treatment with the anti-inflammatory drugs Tobradex or Celecoxib inhibited tumor growth in CLND mice in such a manner that tumor nodule size was comparable to the sham control. Further work will be necessary to support the therapeutic use of these drugs by testing, for example, their effect on tumor progression in non-surgery RET mice.

Dexamethasone is one of the active ingredients of Tobradex and is a synthetic analogue of glucocorticoid, which down-regulates the expression of pro-inflammatory genes including TNF-α [46], IL-1β [47, 48], cox-2 [48, 49], and inducible nitric oxide synthase [50]. Hence, the effect of Dexamethasone on CLND-induced tumor growth is likely attributed to its anti-inflammatory effect. In addition to its analgesic property, Celecoxib has anti-tumor effects by blocking angiogenesis and inducing cell cycle arrest and apoptosis [51, 52]. Although cox-2 is a selective target of celecoxib and its expression has been reported in several cancers including colorectal, breast, pulmonary, prostate and malignant melanoma [31–33], many celecoxib anti-tumor effects are independent of cox-2. In our current work, we find that celecoxib treatment was associated with reduced pAKT levels and decreased anti-apoptotic gene expression. Furthermore, decreased expression of VEGF-A and VEGFR-1 was also observed after treatment in CLND mice. Since cox-2 expression was not detected in the uveal tumor cells from RET mice and PGE_2_ levels remained unchanged in CLND mice regardless of celecoxib treatment, it is likely that celecoxib exerts its anti-tumor effects in our model via a cox-2 independent pathway. Similarly, we observe that Tobradex was associated with reduced expression of pAKT and anti-apoptotic genes. Altogether, these data suggest that both drugs may reverse CLND effect on primary tumor growth in RET mice by interfering with the activation state of the kinase AKT and the balance between anti-apoptotic and pro-apoptotic genes.

Surgical resection of tumors is often followed by regrowth at the primary site and outgrowth of metastases and several mechanisms have been implicated in mediating these postsurgical effects. Retrospective database analyses identified an early-late bimodal cancer recurrence pattern [53–57] where the first peak of recurrence appears to be closely associated with the tumor resection itself. While surgery remains a standard cancer treatment, the perioperative period represents a high risk of metastases development that result from the modulation of tumor dormancy [58]. Surgical stress responses can lead to immune suppression by reducing natural killer cell function, and promoting Th2 responses and tumor associated macrophages (TAM) infiltration [59]. This immunosuppressive environment provides optimal conditions for tumor outgrowth. In the presence of the primary tumor, metastatic growth is suppressed by circulating angiogenic inhibitors such as angiostatin [60]. Upon primary tumor removal, inhibitor levels fall, leading to vigorous expansion of previously dormant metastases [21, 60]. Surgery alone has also been shown to trigger the production of several factors in the wound fluid including TGF-β, bFGF [61], HB-EGF, PDGF [62], as well as oxidative stress [63] and hypoxia [64] leading to the establishment and rapid outgrowth of metastases. Interestingly, we showed previously in the same RET mouse model that surgical removal of eye tumors resulted in increased local and distant tumor growth and such growth was associated with increased TAM density [65]. Similarly, in the transplanted tumor models using Lewis lung carcinoma, T241 sarcoma and B16F10 melanoma, removal of the primary tumor led to large and highly neovascularized growing metastases and reduced apoptosis in metastatic tumor cells [21]. Therefore, by analogy to surgical resection of tumor, we speculate that the surgical trauma associated with LN removal may also promote the growth of the primary tumor and accelerate the outgrowth of already-disseminated metastases. However, the fact that tumor growth and cancer cell dissemination were similar in non-surgery and sham RET mouse groups and were only enhanced after surgical resection of LN suggests that the extent of surgical manipulation in sham control group may not trigger significant changes in the microenvironment of primary and disseminated tumor cells. Indeed, a study in lung metastasis mouse model reported that increasing degree of surgical stress augments tumor metastasis [66]. Notably, perioperative administration of NSAID ketorolac, a common surgical anti-inflammatory analgesic, in breast cancer patients, is associated with superior disease-free survival in the first few years after surgery and the absence of early cancer recurrence [67]. Therefore, it is likely that Tobradex and celecoxib may have similar effect in our model. In summary, all these experimental and clinical reports suggest that transient systemic inflammation accompanying surgery may enhance metastasis and may be effectively blocked by peri-surgical anti-inflammatory agents such as ketorolac, Celecoxib and Tobradex.

Finally, although this point will need further investigations, we cannot exclude that a reduction in anti-tumor immunity as a result of the removal of LN, a central site for T cell priming, may account for the enhanced tumor growth and cancer cell dissemination observed in RET mice after lymphadenectomy [15, 16].

Here, we show that CLND enhances primary tumor growth and metastasis in a mouse model of melanoma in part by promoting a pro-tumoral inflammatory environment. Treatment with anti-inflammatory drugs such as celecoxib and Tobradex was beneficial in controlling tumor progression after CLND and warrants further consideration for implementation in the clinics. In sum, this study not only sheds some light on the role of LN in metastasis which is still currently debated, particularly in the clinical setting, but also further highlights the impact of tumor homeostasis on disease development and progression.

## MATERIALS AND METHODS

### Mice

Animal care and experimental procedures were approved by the Singapore IACUC under protocol 120742. MT/RET mice were generated as previously described [14, 15]. For lymphangiography of TDLNs, 10 μl of 12.5 mg/ml FITC-conjugated dextran (MW 2,000,000, Invitrogen) was injected intra- or peri-ocularly followed by visualization under a fluorescent dissecting microscope. Male and female mice at 2–3 weeks of age were subjected to CLND and were euthanized and necropsied after 4 and 21 weeks. Metastases were detected under dissecting microscope during necropsy, removed and photographed with a ruler in the visual field. Tumor areas were measured using ImageJ software (http://rsb.info.nih.gov/ij). Eye tumors were either fixed with formalin or 2% paraformaldehyde (PFA) and 30% sucrose overnight at 4°C, followed by embedding in paraffin or Optimal Cutting Temperature for immunohistochemistry, respectively.

### Surgical procedures

Mice were injected subcutaneously (s.c) with Buprenorphine (0.2–1mg/kg) and Enrofloxacin (20mg/kg) prior to surgery. Mice were anaesthetized with isoflurane to remove hair at the neck area and during the entire surgical procedure. Followed by a skin incision at the neck region, bilateral mandibular and parotids LNs were surgically resected while similar procedure without removing the LNs was performed in the sham control group. Skin incision was closed by interrupted suture and mice were allowed to recover on a heating pad. Following surgery, Buprenorphine and Enrofloxacin were administered s.c for three days.

### Drug treatment

Tobradex (Alcon) was topically applied on the eyes immediately after surgery and daily application continued for 4 weeks. Celecoxib was purchased from LC Laboratories and incorporated into Harlan chow diet 2918. RET mice received 1500ppm Celecoxib chow diet for 4 or 21 weeks after surgical procedures.

### Cell lines and tumor cell isolations

Melan-ret cells [27] were cultured in RPMI-1640 medium (RPMI) supplemented with 5% heat-inactivated fetal bovine serum, 1% non-essential amino acid, 1% sodium pyruvate, 100 U/ml penicillin and 100 μg/ml streptomycin (all from Invitrogen), at 37°C in a humidified atmosphere of 95% air/5%CO2. Primary tumor cells were isolated by digesting tumors with 1 mg/ml Collagenase A and 0.1 mg/ml DNase I (Roche) in RPMI for 30 min at 37°C.

### Flow cytometry analysis

Flow cytometric analysis of uveal, skin, reproductive tract tumor and Melan-ret cell suspension stained for CD45, CD31, PDGFRα and VEGF-A allowed differentiation and quantification of tumor cells expressing VEGF-A. Antibodies used included the following: rat anti-mouse CD31 (Serotec) detected with anti-rat-Alexafluor488 (Invitrogen), APC-conjugated anti-mouse PDGFRα (eBioscience) and PCPCy5.5 –conjugated anti-mouse CD45 (BD Biosciences). For detection of VEGF-A expression in tumor cells, surface antigens staining were first performed as detailed above. Intracellular staining for VEGF-A was then performed with the BD Fixed/Permeabilization Kit^®^, using rabbit purified anti-VEGF-A (Santa Cruz) antibody and revealed with PE-conjugated anti-rabbit IgG (Biolegend).

### Immunohistochemistry

Formalin-fixed paraffin-embedded uveal tumor sections (10 μm) were immunolabeled with Rabbit anti-S100B (DAKO) and anti-CD31 (Biolegend) antibodies to identify melanoma cells and blood endothelial cells, respectively, as described previously [16]. For analysis of proliferating cells, blood and LVs in uveal tumors, cryosections were stained with rat anti-Ki67-biotinalyted (eBioscience), armenian hamster anti-CD31 (PECAM-1, Millipore) or rat anti-CD31 (PECAM-1, BD Biosciences) and rabbit anti-Lyve-1 (Abcam) antibodies. Cy3-conjugated streptavidin, Alexafluor647-conjugated or Cy3-conjugated anti-armenian hamster and Alexafluor488-conjugated anti-rabbit (Jackson ImmunoResearch Laboratories) antibodies were used for detection. Sections were counterstained with 4,6-diamidino-2-phenylindole (DAPI) for cell nuclei visualization and mounted for analysis. Immunofluorescent labelled specimens were viewed with a fluorescence wide field (Axio Imager.Z1, Axioxam HRM camera; Carl Zeiss Micro Imaging, Jena, Germany). Tumor areas and BV size were measured using ImageJ software.

### Quantitative real-time PCR

Total RNA from eyes and LNs was homogenized and extracted using TRIzol reagent (Qiagen) and NucleoSpin RNA II kit (Macherey-Nagel) or Qiagen RNeasy Micro kit. cDNA was reverse transcribed (Roche Applied Biosystem reagents) from 2 μg of RNA and analysed by quantitative PCR with SYBR green (Bio-Rad) with specific primers. The primers sequences are shown in Supplementary Table S1 (see Supplementary Material).

### ELISA and immunoblotting

Tumor-bearing eyes were harvested and homogenized in RIPA buffer (Sigma Chemicals) with a protease inhibitors mixture (Roche Diagnositics) and Phosphatase Inhibitor Cocktail (Calbiochem). For ELISA, Homogenates were centrifuged for 10 min at 4°C at 14,000 x g and supernatants were assayed using commercial VEGF-A (R&D Systems) and PGE_2_ (Cayman Chemical) ELISA kits according to manufacturers' protocols. For immunoblotting, total proteins were resolved by 10 or 12% SDS-PAGE gel, transferred to nitrocellulose membranes and blocked with 5% milk in 0.05% Tween20 in PBS. The antibodies used for immunoblotting recognized cox-2 (Novus Biological), phospho-ERK, total ERK, phospho-AKT^ser473^, AKT and actin (Cell Signalling Technology). Where required, blots were stripped in stripping buffer (Thermo Scientific) at room temperature for 7–10 min for re-blotting for total ERK1/2 and AKT. SuperSignal West Pico or Femto chemiluminescent substrates (Thermo Scientific) were used for ECL and visualized on ChemiDoc XRS (Biorad). ImageJ software was used for Western blot densitometry analysis.

### Statistical analysis

Statistical analysis was performed with Graphpad Prism version 5.0 (GraphPad Software). The tests applied are indicated in the figure legends. Data were presented as mean ± SEM and were statistically analysed by nonparametric Mann-Whitney *U* test or two-tailed *t* test for comparisons between two groups; one-way ANOVA was used to compare multiple groups with one experimental parameter. A *p* value of less than 0.05 was considered to be statistically significant.

## SUPPLEMENTARY FIGURES AND TABLE


