# Supplementary material for: Lymphadenectomy promotes tumor growth and cancer cell dissemination in the spontaneous RET mouse model of human uveal melanoma
Source: Oncotarget. 2015 Nov 2;6(42):44806–18. doi: 10.18632/oncotarget.6326 (PMC4792593; doi:10.18632/oncotarget.6326)
Supplement: Supplementary file 1 [file oncotarget-06-44806-s001.pdf]

SUPPLEMENTARY FIGURES AND TABLE

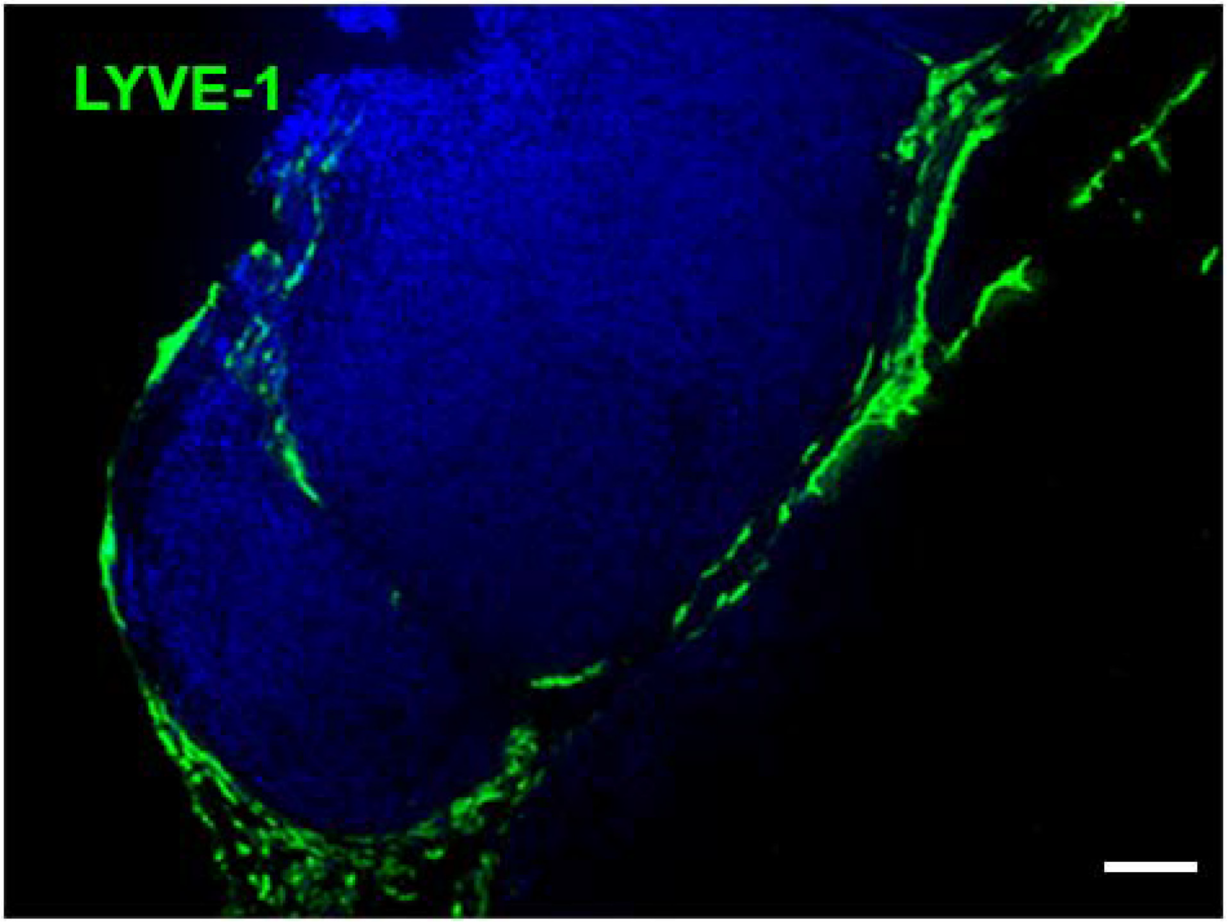

Supplementary Figure S1: Lymphatic vessel distribution in uveal tumor. LV was identified by immunostaining with anti-LYVE-1 antibody (green) on primary tumor-bearing eye cross-sections.

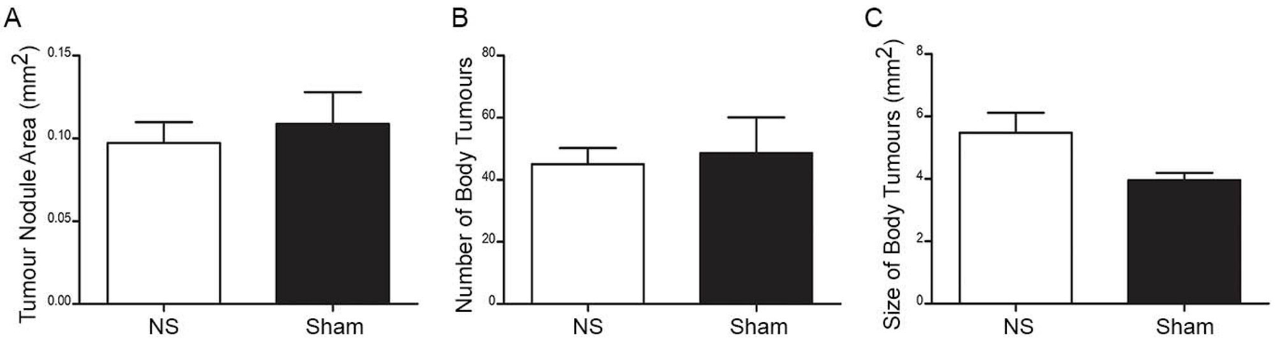

Supplementary Figure S2: A. Comparison of the area of primary tumor nodules between NS and sham control; two-tailed Mann-Whitney test. The number **B.** and size **C.** of body metastases in NS and sham control groups were compared; two-tailed Mann-Whitney.

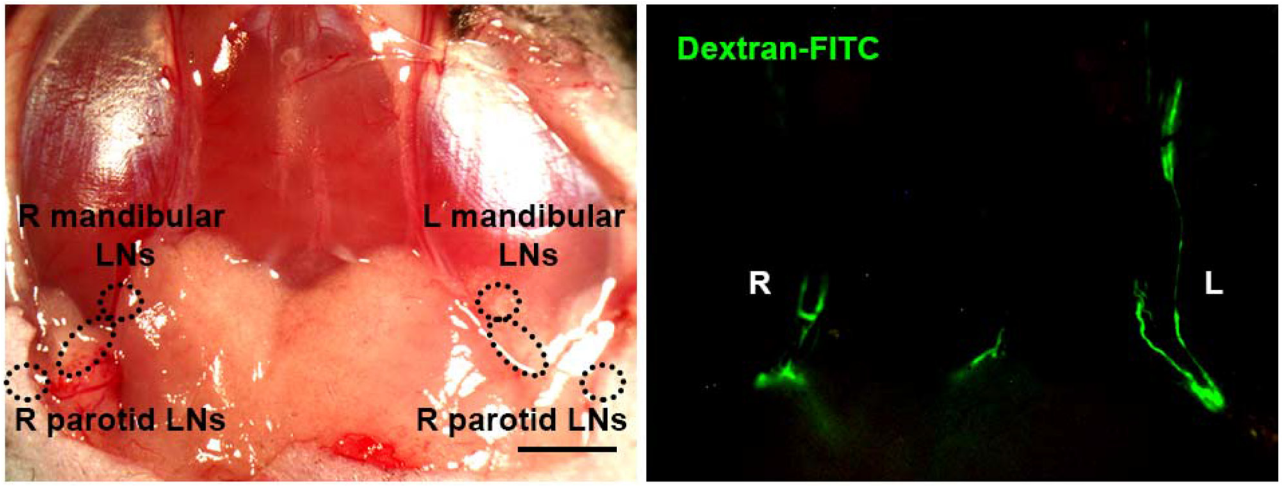

Supplementary Figure S3: Left panel: Macroscopic image of cervical region whereby bilateral mandibular and parotid LN resection was performed. Dotted line represents the location of both mandibular and parotid LNs. Right panel: Intra- and peri-ocular injection with FITC-conjugated dextran verified successful CLND. * shows absence of LV reconnection.

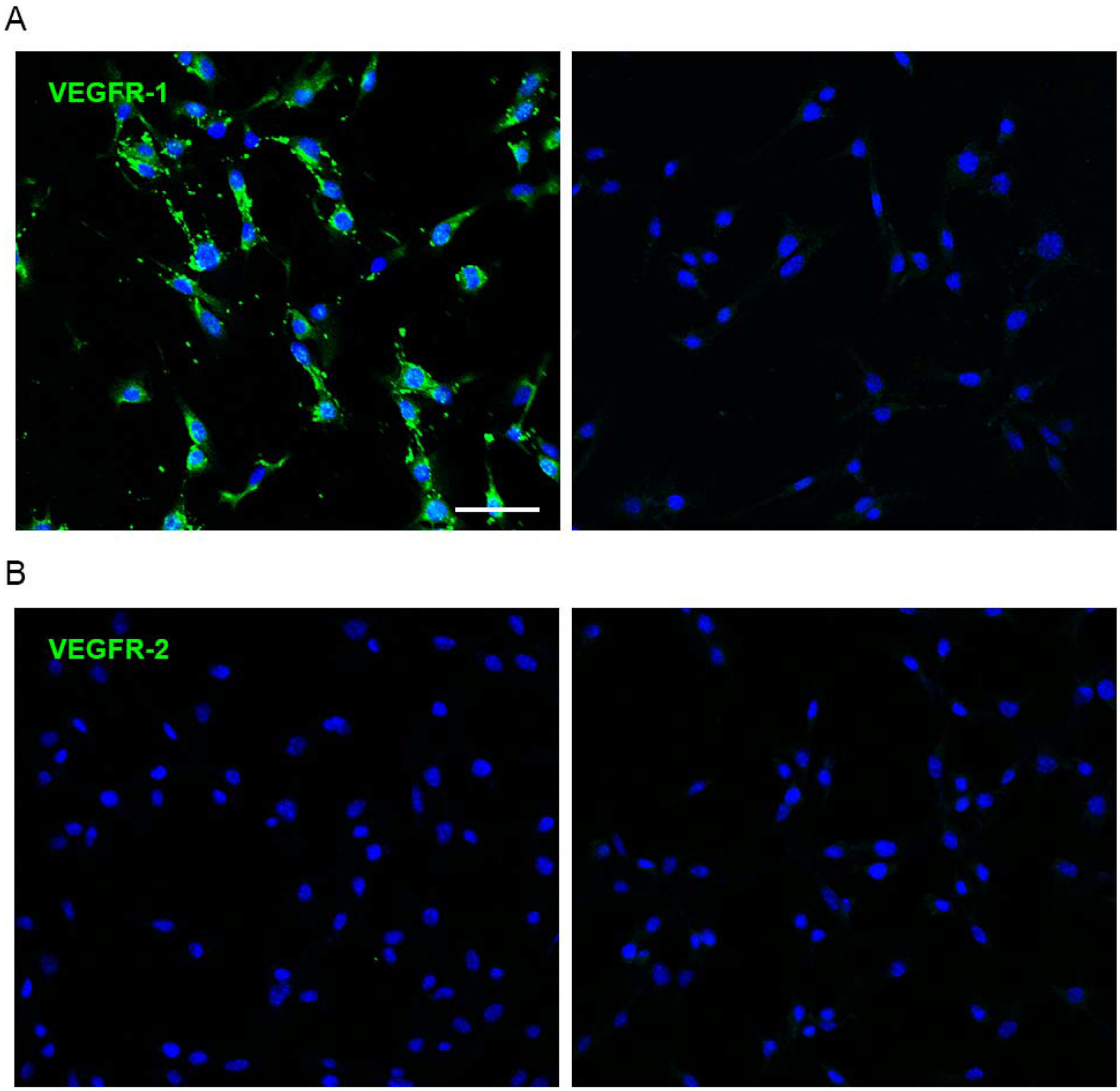

Supplementary Figure S4: Immunofluorescence staining of Melan-ret cells with anti-VEGFR-1 A. or VEGFR-2 B.

Supplementary Table S1: Primers for qPCR

Gene name	Primer sequence
Dct	F: 5′-CTTCCTGAATGGGACCAATG-3′
	R: 5′-ACGGCGTAATTGTAGCCAAG-3′
Prox-1	F: 5′- CTCTCCATCACCAGGGATTG-3′
	R: 5′-CCTTGTAAATGGCCTTCTTCC-3′
VEGF-A	F: 5′- CAGAAGGAGAGCAGAAGTCC-3′
	R: 5′-CTCCAGGGCTTCATCGTTA -3′
VEGFR-1	F: 5′- GAGGAGCTTTCACCGAACTC-3′
	R: 5′- AGCTGGAGAAGCAGAAGCTG-3′
VEGFR-2	F: 5′- TGCCTACCTCACCTGTTTCC-3′
	R: 5′-TCTGTCTGGCTGTCATCTGG-3′
BCL-2	F: 5′-GAGTTCGGTGGGGTCATGTG-3′
	R: 5′-ATAGTTCCACAAAGGCATCCCAG-3′
BCL-XL	F: 5′-CTCCTTTGGCGGGGCACTGTG-3′
	R: 5′-CAAAAGTGTCCCAGCCGCCGTT-3′
Bax	F: 5′-CTTCAACTGGGGCCGCGTGGT-3′
	R: 5′-AGTAGGAGAGGAGGCCTTCCCAGCC-3′
Bak-1	F: 5′-GGCTGCGATGAGTCCCCGTC-3′
	R: 5′- CCACCTGACCCAAGATGCTGTTGG-3′
MCL-1	F: 5′-TGTAAGGACGAAACGGGACT-3′
	R: 5′-AAAGCCAGCAGCACATTTCT-3′
GAPDH	F: 5′-TGCGACTTCAACAGCAACTC-3′
	R: 5′-ATGTAGGCCATGAGGTCCAC-3′
